# Platelet-Rich Plasma Peptides: Key for Regeneration

**DOI:** 10.1155/2012/532519

**Published:** 2012-02-22

**Authors:** Dolores Javier Sánchez-González, Enrique Méndez-Bolaina, Nayeli Isabel Trejo-Bahena

**Affiliations:** ^1^Subsección de Biología Celular y Tisular, Escuela Médico Militar, Universidad del Ejército y Fuerza Aérea, 11200 México City, MEX, Mexico; ^2^Sociedad Internacional para la Terapia Celular con Células Madre, Medicina Regenerativa y Antienvejecimiento S.C. (SITECEM), 53840 Naucalpan, MEX, Mexico; ^3^Facultad de Ciencias Químicas, Universidad Veracruzana, 94340 Orizaba, VER, Mexico; ^4^Centro de Investigaciones Biomédicas-Doctorado en Ciencias Biomédicas, Universidad Veracruzana, 91000 Xalapa, VER, Mexico; ^5^Área de Medicina Física y Rehabilitación, Hospital Central Militar, 11200 México City, MEX, Mexico

## Abstract

Platelet-derived Growth Factors (GFs) are biologically active peptides that enhance tissue repair mechanisms such as angiogenesis, extracellular matrix remodeling, and cellular effects as stem cells recruitment, chemotaxis, cell proliferation, and differentiation. Platelet-rich plasma (PRP) is used in a variety of clinical applications, based on the premise that higher GF content should promote better healing. Platelet derivatives represent a promising therapeutic modality, offering opportunities for treatment of wounds, ulcers, soft-tissue injuries, and various other applications in cell therapy. PRP can be combined with cell-based therapies such as adipose-derived stem cells, regenerative cell therapy, and transfer factors therapy. This paper describes the biological background of the platelet-derived substances and their potential use in regenerative medicine.

## 1. Introduction

Platelets are nonnuclear cellular fragments derived from megakaryocytes in the bone marrow; they are specialized secretory elements that release the contents of their intracellular granules in response to activation. They were discovered by Bizzozero in the 19th century [1] and after Wright observed that megakaryocytes are platelet precursors [2]. Actually we know that platelets synthesize proteins and that pattern of peptides synthesis changes in response to cellular activation [3].

Platelets contain a great variety of proteins molecules, among which are the high presence of signaling, membrane proteins, protein processing, cytoskeleton regulatory proteins, cytokines, and other bioactive peptides that initiate and regulate basic aspects of wound healing [3]. It is known, through efforts such as the platelet proteome project, that more than 300 proteins are released by human platelets in response to thrombin activation [4]. Proteome platelet includes 190 membrane-associated and 262 phosphorylated proteins, which were identified via independent proteomic and phospho proteomic profiling [5].

When platelets fall precipitously below critical levels (usually under 10,000 to 20,000 per cubic millimeter), molecular disassembly opens the zippers formed by adjacent intercellular endothelial junctions, causing extravasation of erythrocytes into the surrounding tissues. In addition to their well-known function in hemostasis, platelets also release substances that promote tissue repair, angiogenesis, and inflammation [6]. Furthermore, they induce the migration and adherence of bone-marrow-derived cells to sites of angiogenesis; platelets also induce differentiation of endothelial-cell progenitors into mature endothelial cells [7].

At the site of the injury, platelets release an arsenal of potent regenerative and mitogenic substances that are involved in all aspects of the wound-healing process including a potential point-of-care biologic treatment following myocardial injury [8]. Based on this, platelet called Platelet-rich Plasma (PRP) has been extensively used for orthopaedic applications; for topical therapy of various clinical conditions, including wounds and soft tissue injuries; and suitable alternative to fetal calf serum for the expansion of mesenchymal stem cells from adipose tissue (see Table 1) [9–12].

In this paper we are going to talk about the platelets, platelet-derived particles, and their biological effects in regenerative medicine.

## 2. Platelets

Platelets are the first element to arrive at the site of tissue injury and are particularly active in the early inflammatory phases of the healing process [6]. They play a role in aggregation, clot formation, homeostasis through cell membrane adherence, and release of substances that promote tissue repair and that influence the reactivity of blood vessels and blood cell types involved in angiogenesis, regeneration, and inflammation [13]. Platelet secretory granules contain growth factors (GFs), signaling molecules, cytokines, integrins, coagulation proteins, adhesion molecules, and some other molecules, which are synthesized in megakaryocytes and packaged into the granules through vesicle trafficking processes [14]. Three major storage compartments in platelets are alpha granules, dense granules, and lysosomes [14].

Platelets mediate these effects through degranulation, in which platelet-derived GF (PDGF), insulin-like GF (IGF1), transforming GF-beta 1 (TGF-*β*1), vascular endothelial GF (VEGF), basic fibroblastic GF (bFGF), and epidermal GF (EGF) are released from alpha granules [15]. In fact, the majority of the platelet substances are contained in alpha granules (see Table 2) [15]. When platelets are activated, they exocytose the granules; this process is mediated by molecular mechanisms homologous to other secretory cells, uniquely coupled to cell activation by intracellular signaling events [16].

Among bioactive molecules stored and released from platelets dense granules are catecholamines, histamine, serotonin, ADP, ATP, calcium ions, and dopamine, which are active in vasoconstriction, increased capillary permeability, attract and activate macrophages, tissue modulation and regeneration. These non-GF molecules have fundamental effects on the biologic aspects of wound healing [5].

For their numerous functions, platelets have developed a set of platelet receptors that are the contact between platelets and their surroundings; they determine the reactivity of platelets with a wide range of agonists and adhesive proteins. Some of these receptors are expressed only on activated platelets [6]. Certain biological mechanisms present in the platelets are shared with other cells, and therefore they contain some common cytoplasmic enzymes, signal transduction molecules, and cytoskeletal components [14].

The platelet lifespan is approximately 7 to 9 days, which they spend circulating in the blood in their resting form. When adhered to exposed endothelium or activated by agonists, they change their shape and secrete the contents of the granules (including ADP, fibrinogen, and serotonin), which is followed by platelet aggregation [7]. Initiation of the signaling event within the platelet leads to the reorganization of the platelet cytoskeleton, which is visible as an extremely rapid shape change [17].

## 3. Platelet-Rich Plasma (PRP)

Platelets are activated either by adhesion to the molecules that are exposed on an injured endothelium, such as von Willebrand Factor (vWF), collagen, fibronectin, and laminin, or by physiologic agonists such as thrombin, ADP, collagen, thromboxane A2, epinephrine, and platelet-activating factors [18].

PRP has been used clinically in humans since the 1970s for its healing properties attributed of autologous GF and secretory proteins that may enhance the healing process on a cellular level [19]. Furthermore, PRP enhances the recruitment, proliferation, and differentiation of cells involved in tissue regeneration [20]. PRP-related products, also known as platelet-rich concentrate, platelet gel, preparation rich in growth factors (PRGF), and platelet releasate, have been studied with in vitro and in vivo experiments in the fields of surgical sciences mainly [21].

Depending on the device and technique used, PRP can contain variable amounts of plasma, erythrocytes, white blood cells, and platelets. The platelet concentration should be increased above baseline or whole blood concentration. It is generally agreed upon that PRP should have a minimum of 5 times the number of platelets compared to baseline values for whole blood to be considered “platelet rich” [22].

This conclusion is supported by in vitro work showing a positive dose-response relationship between platelet concentration and proliferation of human mesenchymal stem cells, proliferation of fibroblasts, and production of type I collagen [23]. This suggests that the application of autologous PRP can enhance wound healing, as has been demonstrated in controlled animal studies for both soft and hard tissues [24, 25].

Autologous PRP represents an efficacious treatment for its use in wound healing like chronic diabetic foot ulceration due to multiple growth factors, is safe for its autologous nature, and is produced as needed from patient blood. Like we said, key for self-regeneration [26].

Upon activation, platelets release their granular contents into the surrounding environment. The platelet alpha granules are abundant and contain many of the GFs responsible for the initiation and maintenance of the healing response [14]. These GFs have been shown to play an important role in all phases of healing. The active secretion of these proteins by platelets begins within 10 minutes after clotting, with more than 95% of the presynthesized GFs secreted within 1 hour. After this initial burst, the platelets synthesize and secrete additional proteins for the balance of their life (5–10 days) [10].

The fibrin matrix formed following platelet activation also has a stimulatory effect on wound healing. The fibrin matrix forms by polymerization of plasma fibrinogen following either external activation with calcium or thrombin or internal activation with endogenous tissue thromboplastin [23]. This matrix traps platelets allowing a slow release of a natural combination of GF while providing a provisional matrix that provides a physical framework for wound stem cells and fibroblast migration and presentation of other biological mediators such as adhesive glycoproteins [27, 28].

PRP with a platelet concentration of at least 1 000 000 platelets/*μ*L in 5 mL of plasma is associated with the enhancement of healing [29]. PRP can potentially enhance healing by the delivery of various GF and cytokines from the alpha granules contained in platelets and has an 8-fold increase in GF concentrations compared with that of whole blood [30].

The use of PRP to enhance bone regeneration and soft tissue maturation has increased dramatically in the fields of orthopedics, periodontics, maxillofacial surgery, urology, and plastic surgery over the last years. However, controversies exist in the literature regarding the added benefit of this procedure. While some authors have reported significant increases in bone formation and maturation rates [21], others did not observe any improvement [31].

The wound-healing process is a complex mechanism characterized by four distinct, but overlapping, phases: hemostasis, inflammation, proliferation, and remodeling [8]. The proliferative phase includes blood vessel formation by endothelial cells and bone synthesis by osteoblasts. All these events are coordinated by cell-cell interactions and by soluble GF released by various cell types. Recent reviews have emphasized the need for additional research aiming to characterize PRP in terms of GF content and their physiological roles in wound healing [32–34].

Thrombin represents a strong inducer of platelet activation leading to GF release [35]. It is also known that particulate grafts, when combined with calcium and thrombin treated PRP, possess better handling characteristics and higher GF content [31]. Typically, thrombin concentrations used in clinical applications vary between 100 and 200 units per mL [21], while platelet aggregation is maximum in the range of 0.5 to 4 units per mL [36].

The basic cytokines identified in platelets play important roles in cell proliferation, chemotaxis, cell differentiation, regeneration, and angiogenesis [28]. A particular value of PRP is that these native cytokines are all present in “normal” biologic ratios. The platelets in PRP are delivered in a clot, which contains several cell adhesion molecules including fibronectin, fibrin, and vitronectin. These cell adhesion molecules play a role in cell migration and thus also add to the potential biologic activity of PRP. The clot itself can also play a role in wound healing by acting as conductive matrix or “scaffold” upon which cells can adhere and begin the wound-healing process [28].

PRP can only be made from anticoagulated blood. Preparation of PRP begins by addition of citrate to whole blood to bind the ionized calcium and inhibit the clotting cascade [15]. This is followed by one or two centrifugation steps. The first centrifugation step separates the red and white blood cells from plasma and platelets. The second centrifugation step further concentrates the platelets, producing the PRP separate from platelet-poor plasma [19].

An important point is that clotting leads to platelet activation, resulting in release of the GF from the alpha granules, otherwise known as degranulation. Approximately 70% of the stored GFs are released within 10 minutes, and nearly 100% of the GFs are released within 1 hour. Small amounts of GF may continue to be produced by the platelet during the rest of its lifespan (1 week) [21].

A method to delay the release of GF is possible by addition of calcium chloride (CaCl_2_) to initiate the formation of autogenous thrombin from prothrombin. The CaCl_2_ is added during the second centrifugation step and results in formation of a dense fibrin matrix. Intact platelets are subsequently trapped in the fibrin matrix and release GF slowly over a 7-day period. The fibrin matrix itself may also contribute to healing by providing a conductive scaffold for cell migration and new matrix formation [27].

## 4. Growth Factors (GFs)

Platelets are known to contain high concentrations of different GF and are extremely important in regenerative process; activation of the platelet by endothelial injury initiates the wound-healing process [30]. When platelets are activated, their alpha granules are released, resulting in an increased concentration of GF in the wound milieu [14].

There is increasing evidence that the platelet cell membranes themselves also play a crucial role in wound healing through their GF receptor sites [28]. GFs are found in a wide array of cells and in platelet alpha granules [37]. Table 1 gives an overview of some of the more extensively studied GFs and their involvement in wound healing. There are many more, both discovered and undiscovered, GFs. The platelet is an extremely important cell in wound healing because it initiates and plays a major role in the wound regenerative process [38].

The first discovered GF was EGF in 1962 by Cohen [39]. It was not until 1989 before clinical trials with EGF were attempted to demonstrate enhanced wound healing. Studies did demonstrate that EGF can accelerate epidermal regeneration and enhance healing of chronic wounds [40].

PDGF was discovered in 1974 and is ubiquitous in the body. It is known to be released by platelet alpha granules during wound healing and stimulate the proliferation of many cells, including connective tissue cells. In fact, thus far, high-affinity cell-surface receptors specific for PDGF have only been demonstrated on connective tissue cells. When released, PDGF is chemotactic for monocytes, neutrophils, and fibroblasts. These cells release their own PDGF, thus creating a positive autocrine feedback loop [41]. Other functions of PDGF include effects on cell growth, cellular migration, metabolic effects, and modulation of cell membrane receptors [42].

PDGFs were first identified as products of platelets which stimulated the proliferation in vitro of connective tissue cell types such as fibroblasts [43].

The PDGF system, comprising four isoforms (PDGF-A, -B, -C, and -D) and two receptor chains (PDGFR-alpha and -beta), plays important roles in wound healing, atherosclerosis, fibrosis, and malignancy. Components of the system are expressed constitutively or inducibly in most renal cells [42]. They regulate a multitude of pathophysiologic events, ranging from cell proliferation and migration to extracellular matrix accumulation, production of pro- and anti-inflammatory mediators, tissue permeability, and regulation of hemodynamics [43].

Inactivation of PDGF-B and PDGF beta receptor (PDGFRb) genes by homologous recombination in embryonic stem cells shows cardiovascular, hematological, and renal defects. The latter is particularly interesting since it consists of a specific cellular defect: the complete loss of kidney glomerular mesangial cells and the absence of urine collection in the urinary bladder [43].

PDGF-C and PDGFR-alpha contribute to the formation of the renal cortical interstitium. Almost all experimental and human renal diseases are characterized by altered expression of components of the PDGF system. Infusion or systemic overexpression of PDGF-B or -D induces prominent mesangioproliferative changes and renal fibrosis. Intervention studies identified PDGF-C as a mediator of renal interstitial fibrosis and PDGF-B and -D as key factors involved in mesangioproliferative disease and renal interstitial fibrosis [43–45].

Fréchette et al., demonstrated that the release of PDGF-B, TGF-beta1, bFGF, and VEGF is significantly regulated by the amount of calcium and thrombin added to the PRP and that PRP supernatants are more mitogenic for endothelial cells than whole-blood supernatants [11]. Other GFs such as epidermal growth factor (EGF), transforming growth factor-alpha (TGF-alpha), insulin-like growth factor-1 (IGF-1), angiopoietin-2 (Ang-2), and interleukin-1beta (IL-1beta) are also known to play important roles in the wound-healing process [28].

In 2008, Wahlström et al., demonstrated that growth factors released from platelets had potent effects on fracture and wound healing. The acidic tide of wound healing, that is, the pH within wounds and fractures, changes from acidic pH to neutral and alkaline pH as the healing process progresses [44]. They investigated the influence of pH on lysed platelet concentrates regarding the release of growth factors. The platelet concentrates free of leukocyte components were lysed and incubated in buffers with pH between 4.3 and 8.6. Bone morphogenetic protein-2 (BMP-2), platelet-derived growth factor (PDGF), transforming growth factor-beta (TGF-beta), and vascular endothelial growth factor (VEGF) were measured by quantitative enzyme-linked immunosorbent assays. BMP-2 was only detected in the most acidic preparation (pH 4.3), which is interesting since BMP-2 has been reported to be an endogenous mediator of fracture repair and to be responsible for the initiation of fracture healing. These findings indicate that platelets release substantial amounts of BMP-2 only under conditions of low pH, the milieu associated with the critical initial stage of fracture healing [44].

Recently, Bir et al., demonstrated stromal cell-derived factor 1-*α* (SDF-1*α*) PRP from diabetic mice. The concentration (pg/mL) of different growth factors was significantly higher in the PRP group than in the platelet-poor plasma (PPP) group. The concentrations (pg/mL) of SDF-1*α* (10,790 ± 196 versus 810 ± 39), PDGF-BB (45,352 ± 2,698 versus 958 ± 251), VEGF (53 ± 6 versus 30 ± 2), bFGF (29 ± 5 versus 9 ± 5), and IGF-1 (20,628 ± 1,180 versus 1,214 ± 36) were significantly higher in the PRP group than in the PPP group, respectively [46].

## 5. Platelet GF as Treatment

### 5.1. In Vitro Studies

Knowledge of GF and their function is far from complete. Many of the known functions were learned through in vitro study. Although many GFs are associated with wound healing, PDGF and TGF-*β*1 appear to be two of the more integral modulators [46]. PDGF has activity in early wound healing (during the acid tide). In vitro studies have shown that at lower pH (5.0), platelet concentrate lysate has increased concentrations of PDGF, with an increased capacity to stimulate fibroblast proliferation [23]. TGF-*β* increases the production of collagen from fibroblasts [47]. Its release in vitro is enhanced by neutral or alkaline pH, which correspond to the later phases of healing [10]. Through modulation of interleukin-1 production by macrophages, PRP may inhibit excessive early inflammation that could lead to dense scar tissue formation [48]. Insulin-like GF-I (IGF-1) has also been extensively studied for its ability to induce proliferation, differentiation, and hypertrophy of multiple cell lines. Separate analyses of GF in PRP have shown significant increases in PDGF, VEGF, TGF-*β*1, and EGF, compared with their concentrations in whole blood [15, 49].

IGF-1 has two important functions: chemotaxis for vascular endothelial cells into the wound which results in angiogenesis and promoting differentiation of several cell lines including chondroblasts, myoblasts, osteoblasts, and hematopoietic cells [50].

TGF-*β* is a member of the newest family of proteins discovered. Two major sources of this protein are the platelet and macrophage. TGF-*β* causes chemotactic attraction and activation of monocytes, macrophages, and fibroblasts. The activated fibroblasts enhance the formation of extracellular matrix and collagen and also stimulate the cells ability to contract the provisional wound matrix [51]. Macrophages infiltration promotes TGF-*β* that induces extracellular matrix such as collagen and fibronectin; however alpha-mangostin prevents the increase in this molecule in rats with Cisplatin-induced nephrotoxicity [52].

### 5.2. In Vivo Studies

In vivo study is much more complex due to the inability to control the environment. A further complexing matter is the fact that the same GF, depending on the presence or absence of other peptides, may display either stimulatory or inhibitory activity within the same cell. Also, a particular GF can alter the binding affinity of another GF receptor [53].

Release of PDGF can have a chemotactic effect on monocytes, neutrophils, fibroblasts, stem cells, and osteoblasts. This peptide is a potent mitogen for mesenchymal cells including fibroblasts, smooth muscle cells and glial cells [54] and is involved in all three phases of wound healing, including angiogenesis, formation of fibrous tissue, and reepithelialization [41].

TGF beta released from platelet alpha granules is a mitogen for fibroblasts, smooth muscle cells, and osteoblasts. In addition, it promotes angiogenesis and extracellular matrix production [41]. VEGF promotes angiogenesis and can promote healing of chronic wounds and aid in endochondral ossification. EGF, another platelet-contained GF, is a mitogen for fibroblasts, endothelial cells, and keratinocytes and also is useful in healing chronic wounds [55].

IGF, another platelet-contained GF regulates bone maintenance and is also an important modulator of cell apoptosis, and, in combination with PDGF, can promote bone regeneration [56].

However, there are conflicting results with regard to IGF-1, where the majority of studies reported no increase in IGF-1 in PRP, compared with whole blood. There are also conflicting results regarding the correlation between the GF content and platelet counts in PRP [57]. The basis of these contradictions is not fully understood and may be related to variability in patient age, health status, or platelet count. Alternatively, differences in GF content and platelet count may be due to the various methods of processing, handling, and storing of samples, in addition to the type of assay performed. The diversity of PRP products should be taken into account when interpreting and comparing results and methods for generating PRP [10].

VEGF, discovered 25 years ago, was initially referred to as vascular permeability factor [58]. In mammals, there are at least four members of the VEGF family: VEGF-A, VEGF-B, and the VEGF-C/VEGF-D pair, which has a common receptor, VEGF receptor 3 (VEGF-R3) [59]. VEGF-A is a proangiogenic cytokine during embryogenesis and contributes to vascular integrity: selective knockout of VEGF-A in endothelial cells increases apoptosis, which compromises the integrity of the junctions between endothelial cells [60, 61]. VEGF-B, which can form heterodimers with VEGF-A, occurs predominantly in brown fat, myocardium, and skeletal muscle [62]. VEGF-C and VEGF-D seem to regulate lymphangiogenesis. The expression of VEGF-R3 in adults is restricted to the lymphatics and fenestrated endothelium [63]. Neuropilin 1 and neuropilin 2 are receptors that bind specific VEGF family members and are important in neuronal development and embryonic vasculogenesis [64].

Megakaryocytes and platelets contain the three major isoforms of VEGF-A; after exposure to thrombin in vitro, they release VEGF-A [65–67]. VEGF-A alters the endothelial-cell phenotype by markedly increasing vascular permeability, upregulating expression of urokinase, tissue plasminogen activator, connexin, osteopontin, and the vascular-cell adhesion molecule [68].

## 6. PRP in Cell Therapy and Regenerative Medicine

PRP can be combined with cell-based therapies such as adipose-derived stem cells, regenerative cell therapy, and transfer factors therapy [69]. While this is a relatively new concept, the strategy is appealing as the regenerative matrix graft delivers a potent trilogy of regenerative cells, fibrin matrix, and GF [15]. The applications are similar to those for PRP alone with the added benefit of regenerative cell enrichment.

Verrier et al., demonstrated that cultures of human mesenchymal stem cell (MSC) supplemented with platelet-released supernatant (PRS) had differentiation towards an osteoblastic phenotype in vitro possibly mediated by bone morphogenetic protein-2 (BMP-2). PRS showed an osteoinductive effect on MSC, as shown by an increased expression of typical osteoblastic marker genes such as collagen I, bone sialoprotein II, BMP-2, and matrix metalloproteinase-13 (MMP-13), as well as by increased Ca^++^ incorporation [70].

Furthermore, the role of platelets in hemostasis may be influenced by alteration of the platelet redox state, the presence of endogenous or exogenous antioxidants, and the formation of reactive oxygen and nitrogen species [71]. As discussed by Sobotková et al., [71], trolox and resveratrol inhibit aggregation of washed platelets and PRP activated by ADP, collagen, and thrombin receptor-activating peptide. Antioxidants, apart from nonspecific redox or radical-quenching mechanisms, inhibit platelet activation also by specific interaction with target proteins. In this context, powerful natural antioxidants, like nordihydroguaiaretic acid (NDGA) extracted from *Larrea tridentata* [72], S-allylcysteine (SAC), the most abundant organosulfur compound in aged garlic extract (AG) [73], sulforaphane (SFN), an isothiocyanate produced by the enzymatic action of myrosinase on glucoraphanin, a glucosinolate contained in cruciferous vegetables [74], and acetonic and methanolic extracts of *Heterotheca inuloides*, can be administered with ample safety margin in patients treated with PRP [75].

## 7. Advantages, Limitations, and Precautions

PRP as autologous procedure eliminates secondary effects and unnecessary risks for chemically processed strange molecules, is a natural reserver of various growth factors that can be collected autologously, and is costeffective [76–81] (see Table 3). Thus for clinical use, no special considerations concerning antibody formation and infection risk are needed. The key of our health and our own regeneration resides in our own body. Nevertheless this treatment is not the panacea, it is only the beginning in this new age of the regenerative medicine. Some clinical devices to automatically prepare PRPs are available at present. PRP are consistently being used clinically in the department of orthopedics and plastic surgery (oral, maxillary facial) for a long time. On the basis of research evidence, some publications have reported positive results in either bone or soft tissue healing. It is recommended to avoid the abuse and use generalized of this procedure before any disease. Until now, their clinical applications still are limited. However, some research concludes that there is no or little benefit from PRP. This is likely due to faster degradation of growth factors in PRP since some authors suggest using sustained release form of PRP to deliver optimal effect of PRP. Gelatin hydrogel is also being used clinically as a slow, sustained release of carrier for growth factors in our center recently [47].

## 8. Conclusions

PRP as therapeutic option is a powerful tool nowadays for the localized delivery of great variety of biologically active GF to the site of injury and is supported by its simplicity, potential cost effectiveness, safety, and permanent availability [11].

Platelet concentrates are potentially useful in wound-healing applications because they function as both a tissue sealant and a drug delivery system that contains a host of powerful mitogenic and chemotactic GFs. However, the method of PRP preparation has a potentially significant impact on the different levels of platelet recovery and activation [10]. Platelet activation during preparation of the platelet concentrate can result in early alpha granule release and loss of the GF during the collection process. It is therefore critical to recognize that each PRP preparation method may differ in regard to platelet number, platelet activation rates, and GF profiles [15].

In this regard, therefore, it is critical to define the extent of platelet activation that occurs during graft preparation. If platelets become activated and release the contents of the alpha granules during the centrifugation process, the GF will be diluted and lost into the plasma. To ensure that platelets are intact until the PRP fraction has been collected, platelet surface marker for platelet activation P selectin can be measured [30]. However, in all methods, the application of PRP is really providing a sufficient dose of these useful bioactive peptides for wound healing and regenerative process [82].

## Figures and Tables

**Table 1:** Peptidic growth factors present in platelet-rich plasma (PRP).

Name	Cytogenetic location	Biologic activities
Transforming growth factor, beta-I; TGFB1	19q13.2	Controls proliferation, differentiation, and other functions in many cell types
Platelet-derived growth factor, alpha polypeptide; PDGFA	7p22.3	Potent mitogen for connective tissue cells and exerts its function by interacting with related receptor tyrosine kinases
Platelet-derived growth factor, beta polypeptide; PDGFB	22q13.1	Promotes cellular proliferation and inhibits apoptosis
Platelet-derived growth factor C; PDGFC	4q32.1	Increases motility in mesenchymal cells, fibroblasts, smooth muscle cells, capillary endothelial cells, and neurons
Platelet-derived growth factor D; PDGFD	11q22.3	Involved in developmental and physiologic processes, as well as in cancer, fibrotic diseases, and arteriosclerosis
Insulin-like growth factor I; IGF1	12q23.2	Mediates many of the growth-promoting effects of growth hormone
Fibroblast growth factor I; FGF1	5q31.3	Induces liver gene expression, angiogenesis and fibroblast proliferation
Epidermal growth factor; EGF	4q25	Induces differentiation of specific cells, is a potent mitogenic factor for a variety of cultured cells of both ectodermal and mesodermal origin
Vascular endothelial growth factor A; VEGFA	6p21.1	Is a mitogen primarily for vascular endothelial cells, induces angiogenesis
Vascular endothelial growth factor B; VEGFB	11q13.1	Is a regulator of blood vessel physiology, with a role in endothelial targeting of lipids to peripheral tissues
Vascular endothelial growth factor C; VEGFC	4q34.3	Angiogenesis and endothelial cell growth, and can also affect the permeability of blood vessels

Includes, name, cytogenetic location, and biologic activities of platelet growth factors. Furthermore, PRP content other proteins like interleukin-8, macrophage inflammatory protein-1 alpha, and platelet factor-4.

**Table 2:** Some bioactive peptides present in the alpha granules of platelets.

General activity categories	Specific molecules	Cytogenetic location	Biologic activities
	Tissue factor pathway inhibitor; TFPI	2q32.1	Regulates the tissue factor-(TF-) dependent pathway of blood coagulation
	Kininogen; KNG	3q27.3	Plays an important role in assembly of the plasma kallikrein
	Growth arrest-specific 6; GAS6	13q34	Stimulates cell proliferation
	Multimerin; MMRN	4q22	Carrier protein for platelet factor V
Clotting factors and related proteins	Antithrombin; AT	1q25.1	Is the most important inhibitor of thrombin
	Protein S; PROS1	3q11.1	Inhibits blood clotting
	Coagulation factor V; F5	1q24.2	Acts as a cofactor for the conversion of prothrombin to thrombin by factor Xa
	Coagulation factor XI; F11	4q35.2	It participates in blood coagulation as a catalyst in the conversion of factor IX to factor IXa in the presence of calcium ions

	Plasminogen; PLG	6q26	Induces plasmin production (leads to fibrinolysis)
	Plasminogen activator inhibitor 1; PAI1	7q22.1	Regulation of plasmin production
	Alpha-2-plasmin inhibitor	17p13.3	Inactivation of plasmin
Fibrinolytic factors and related proteins	Osteonectin; ON	5q33.1	Inhibits cell-cycle progression and influences the synthesis of extracellular matrix (ECM)
	Histidine-rich glycoprotein; HRG	3q27.3	Interacts with heparin and thrombospondin
	Thrombin-activatable fibrinolysis inhibitor; TAFI	13q14.13	Attenuates fibrinolysis
	Alpha-2-Macroglobulin; A2M	12p13.31	Carrier of specific growth factors and induces cell signaling

	Tissue inhibitor of metalloproteinase 4; TIMP4	3p25.2	Inhibits matrix metalloproteinases (MMPs), a group of peptidases involved in degradation of the extracellular matrix
	Complement component 1 inhibitor; C1NH	11q12.1	Inhibits serine proteinases including plasmin, kallikrein, and coagulation factors XIa and XIIa
Proteases and antiproteases	Alpha-1-antitrypsin (serpin peptidase inhibitor)	14q32.13	Acute phase protein, inhibits a wide variety of proteases and enzymes
	Nexin 2; SNX2	5q23.2	Modulates intracellular trafficking of proteins to various organelles

	Platelet factor 4; PF4	4q13.3	Inhibition of angiogenesis
Basic proteins	*β*-thromboglobulin (Pro-platelet basic protein; PPBP)	4q13.3	Platelet activation, inhibition of angiogenesis
	Endostatin (Collagen, type XVIII, Alpha-1; COL18A1)	21q22.3	Inhibitors of endothelial cell migration and angiogenesis

	Fibrinogen; FG	4q31.3	Blood clotting cascade (fibrin clot formation)
	Fibronectin; FN	2q35	Binds to cell-surface integrins, affecting cell adhesion, cell growth, migration, and differentiation
Adhesive proteins	Vitronectin; VTN	17q11.2	Induces cell adhesion, chemotaxis
	Thrombospondin I; THBS1	15q14	Inhibition of angiogenesis
	Laminin-8	18p11.31-p11.23	Modulates cell contact interactions

It is described general activity categories, specific molecules, cytogenetic location and biologic activities. Furthermore, alpha granules include growth factors of Table 1, membrane glycoproteins, and others proteins like albumin and immunoglobulins.

**Table 3:** Platelet-plasma-derived peptides are current in clinical use and clinical trials.

Year	Researchers	Health problems	Clinical protocols	Level of evidence	Results
2005	Carreon et al.	Bone healing in instrumented posterolateral spinal fusions	Retrospective cohort study to evaluate rates of nonunionin patients (*n *= 76) with autologous iliac bone graft augmented with platelet gel	Level 4, case control group of 76 randomly selected patients who were matched and grafted with autogenous iliac bone graft with no platelet gel	Nonunion rate in platelet gel group was 25%; 17% in control group (*P* = .18)

2006	Mishra and Pavelko	Chronic elbow tendinitis	Cohort, 15 patients injected with PRP	Level 2, 5 controls	Decreased pain at 2 years (measured by visual analog pain score)
	Savarino et al.	Bone healing in varus HTOs for genu varus	Randomized case control, 5 patients with bone grafted with bone chips and PRP	Level 4, 5 controls bone grafted without PRP	No functional or clinical difference; histology shows increased amounts of osteoid and osteoblasts in PRP group

	Sánchez et al.	Achilles tear healing	Case control, 6 repairs with PRP	Level 3, 6 matched retrospective controls	Improved ROM and early return to activity with PRP by ± 4–7 weeks
2007	Dallari et al.	Bone healing in varus HTOs for genu varus	Prospective randomized control: group A, bone chips with platelet gel (*n* = 11); group B, bone chips, BMC, and platelet gel (*n* = 12)	Level 1, 10 controls treated with bone chips only	Biopsies at 6 weeks after surgery showed increased osteoid and osteoblasts in groups A and B; radiographic differences decreased with time; no clinical difference at 1 year among groups
	Kitoh et al.	Bone healing in distraction osteogenesis for limb lengthening and short stature	Retrospective, comparison case control; at 3 weeks, patients injected with expanded BMC with or without PRP (*n* = 32 bones)	Level 3, 60 bones in retrospective control group (high % of congenital etiologies versus PRP group)	Average healing in BMC + PRP was 34 ± 4 d/cm; control group average was 73.4 ± 27 d/cm (*P* = .003)

2009	Sánchez et al.	Bone healing in nonunions	Retrospective, case series; 16 nonhypertrophic nonunions treated with either surgery and PRGF or percutaneous injections of PRGF to stimulate (*n* = 3) without surgery	Level 4, no control group	84% healed after surgical treatment; unclear if PRGF made a difference

Some published human clinical orthopaedic PRP studies. PRP: platelet-rich plasma; ROM: range of motion; HTO: high tibial osteotomy; BMC: bone marrow cells; PRGF: preparation rich in growth factors [27]. Taken from Foster et al. [27].
